# Prevalence and risk factors of schistosomiasis among primary school children in four selected regions of The Gambia

**DOI:** 10.1371/journal.pntd.0009380

**Published:** 2021-05-11

**Authors:** Ebrima Joof, Abdoulie M. Sanyang, Yaya Camara, Alhagie Papa Sey, Ignatius Baldeh, Sharmila Lareef Jah, Serign Jawo Ceesay, Sana M. Sambou, Saikou Sanyang, Christopher M. Wade, Bakary Sanneh

**Affiliations:** 1 National Public Health Laboratories, Ministry of Health and Social Welfare, Banjul, The Gambia; 2 School of Life Sciences, University of Nottingham, Nottingham, United Kingdom; 3 Epidemiology and Disease Control Department, Ministry of Health and Social Welfare, Banjul, The Gambia; 4 World Health Organisation, Country Office, Banjul, The Gambia; 5 Medical Research Council, Atlantic Boulevard, Fajara, The Gambia; 6 Faculty of Business and Management, Arden University, London, United Kingdom; Chinese Center for Disease Control and Prevention, CHINA

## Abstract

**Background:**

The Gambia initiated a control programme for schistosomiasis in 2015. In light of this, recent and comprehensive data on schistosomiasis is required to effectively guide the control programme. This study aimed to evaluate the prevalence and associated risk factors of schistosomiasis among primary school children in The Gambia.

**Methods:**

We utilised data from a previous study conducted in 2015 in 4 regions of The Gambia: North Bank Region (NBR), Lower River Region (LRR), Central River Region (CRR) and Upper River Region (URR). In the parent study, ten schools were selected randomly from each region. Urine and stool samples collected from 25 boys and 25 girls (7–14 years) in each school were examined for urinary schistosomiasis (*Schistosoma haematobium* infection) and intestinal schistosomiasis (*Schistosoma mansoni* infection) using urine filtration, dipstick and Kato-Katz methods.

**Principal findings:**

Urinary schistosomiasis had an overall prevalence of 10.2% while intestinal schistosomiasis had a prevalence of 0.3% among the sampled school children. Prevalence of urinary schistosomiasis was significantly different among regions (χ 2 = 279.958, df = 3, p < 0.001), with CRR (27.6%) being the most endemic region, followed by URR (12.0%), then LRR (0.6%), and NBR (0.0%). Prevalence of intestinal schistosomiasis was also significantly variable among regions, with 4 of the 5 positive cases detected in CRR and 1 case in URR. Every school sampled in CRR had at least one student infected with *S*. *haematobium*, 50% of schools in URR had S. *haematobium* infection, and just one school in LRR had *S*. *haematobium* infection. While *S*. *haematobium* infection was significantly higher in boys (χ 2 = 4.440, df = 1, p = 0.035), no significant difference in infection rate was observed among age groups (χ 2 = 0.882, df = 2, p = 0.643). Two of the 5 students infected with *S*. *mansoni* were boys and 3 were girls. Four of these 5 students were in the 10–12 years age group and 1 was in the 7–9 years age group. Macrohaematuria and microhaematuria were found to be statistically associated with presence of *S*. *haematobium* eggs in urine. Being a male was a risk factor of *S*. *haematobium* infection. Bathing, playing and swimming in water bodies were found to pose less risk for *S*. *haematobium* infection, indicating that the true water contact behaviour of children was possibly underrepresented.

**Conclusion:**

The findings of this study provide invaluable information on the prevalence of schistosomiasis in The Gambia. This was useful for the schistosomiasis control efforts of the country, as it guided mass drug administration campaigns in eligible districts in the study area. More studies on *S*. *mansoni* and its intermediate snail hosts are required to establish its true status in The Gambia. As children sometimes tend to provide responses that potentially please the research or their teacher, data collection frameworks and approaches that ensure true responses in studies involving children should be devised and used.

## Author summary

In this study, we utilised data collected from a previous study that investigated schistosomiasis among primary school children in four regions (NBR, LRR, CRR and URR) of The Gambia, to assess the prevalence and risk factors of the disease. Our analysis revealed that urinary schistosomiasis was the most prevalent (10.2%) form of the disease and intestinal schistosomiasis was very rare (0.3%) among the school children. Urinary schistosomiasis was found in all study regions except NBR. CRR had the highest prevalence of urinary schistosomiasis and all the schools sampled in the region had at least on student with the infection. Fifty percent of schools in URR had urinary schistosomiasis infection and just one school in LRR had the infection. Presence of blood in urine was a good indicator of urinary schistosomiasis. While being male increases the chance of getting urinary schistosomiasis, playing, bathing and swimming in water bodies unexpedly turned out to pose less risk for infection, indicating the true water contact activities of the children were probably underrepresented in the data. The study provides vital information for the schistosomiasis control efforts of The Gambia. Further studies are required to know the real status of intestinal schistosomiasis in the country.

## Introduction

Schistosomiasis is a parasitic neglected tropical disease that affects nearly 192 million people in over 40 countries in Sub-Saharan Africa [1]. It is caused by flatworms of the genus *Schistosoma*. Of the six *Schistosoma* species known to cause disease in humans [2], two are common in Sub-Saharan Africa: *Schistosoma haematobium*, the causative agent of urogenital schistosomiasis and *Schistosoma mansoni*, the causative agent of intestinal schistosomiasis [3]. Approximately two-thirds of all schistosomiasis cases in Sub-Saharan Africa are caused by *S*. *haematobium* [4,5]. Both *S*. *haematobium* and *S*. *mansoni* use molluscan snails as intermediate hosts to reach the infective larval stage in freshwater bodies, which become sources of infection to humans that come in contact with these sites [6,7].

Water contact activities such as playing, bathing, swimming, and irrigation farming have been found to predispose people to schistosome infections, and male children are reported to be at higher risk of being infected than female children [8–11]. As school age children are more likely to make contact with these freshwater bodies, they have an increased risk of getting infected with schistosomiasis [12,13]; hence most studies target this age group. The WHO recommended methods for mapping and field diagnosis of schistosomiasis are the urine filtration technique for detecting *S*. *haematobium* eggs in urine [14,15] and the Kato-Katz thick smear method [16] for detecting *S*. *mansoni* eggs in stool [2]. Urine dipstick is also often used alongside the filtration technique to detect blood in urine (haematuria), which is a good indicator of *S*. *haematobium* infection [13,17].

Since the London Declaration, made on the 30th January 2012, which called for the control and elimination of schistosomiasis [18,19], The Gambia has initiated a neglected tropical diseases (NTDs) control programme for schistosomiasis. However, there was a lack of current and comprehensive information on the prevalence and endemicity of schistosomiasis in the country to effectively guide this control programme. Some early studies on schistosomiasis in The Gambia focused on prevalence and distribution of the disease in some communities in the country [20,21] while others looked at its transmission and vectors [22,23]. The later studies shifted from observational to more interventional approaches with one study [24] using mollusciciding to drastically reduce *Bulinus senegalensis* snail populations in seasonal pools, such that transmission to humans was halted for three years in the intervention communities. As the latest of these studies were over three decades ago, and the current clinical data on schistosomiasis from hospitals was neither reliable nor adequate [25], there is a need for more up to date and inclusive information on the endemicity of schistosomiasis to effectively guide control strategies. In light of the above, this study evaluated the prevalence and associated risk factors of schistosomiasis in four selected regions of The Gambia.

## Methods

### Ethics statement

Ethical approval was obtained for the parent study [26] and informed consent was obtained from students and their parents and guardians. Written consent forms were provided and signed by heads of schools on behalf of all students whose parents, guardians or carers gave consent, after being contacted by the heads of schools prior to the study exercise. Furthermore, information about the study was provided by the data collection teams to each of the participants—after which informed verbal consent was obtained from each student as they were enrolled in the study.

### Data source

**Fig 1:**
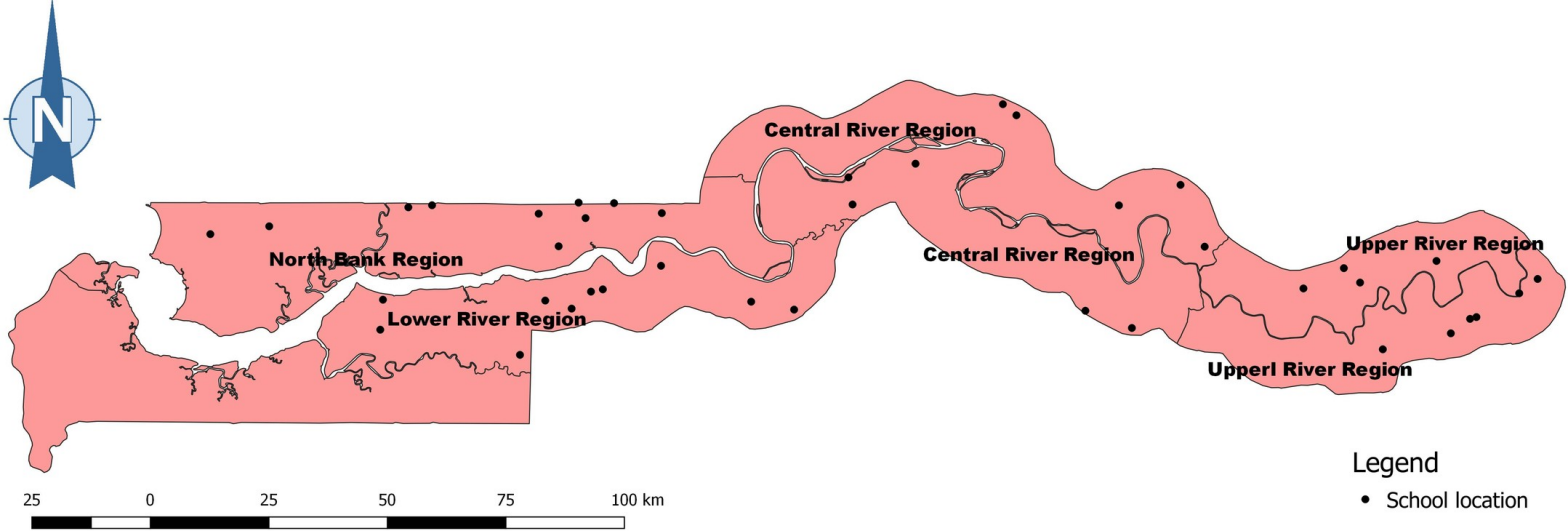
A map of The Gambia showing the locations of the 40 schools sampled across the 4 regions of the country. **Link to map base layer used in creating this figure:** https://data.humdata.org/dataset/gambia-administrative-boundaries.

In this study, we extracted and utilised data from the following study: Field evaluation of a schistosome circulating cathodic antigen rapid test kit at point-of-care for mapping of schistosomiasis endemic districts in The Gambia [26], which was conducted as a sub study during The Gambia NTD mapping survey [27] in 2015. The study [26] was prospective and cross-sectional in design. The investigations were carried out in 4 regions of The Gambia in 2015: North Bank Region (NBR), Lower River Region (LRR), Central River Region (CRR), and Upper River Region (URR). Ten schools were selected at random from each region for the study (Fig 1). Following the WHO guidelines for participant selection in schistosomiasis mapping studies, 50 students (25 boys and 25 girls) between the ages of 7 and 14 years were randomly selected from each school. However, in some schools more than 50 students were sampled to make up for lost or dodgy samples. The total number of students sampled was 2,018 students distributed as follow; 501 in NBR, 500 in LRR, 515 in CRR, and 502 in URR [26]. The sample size determination followed the WHO recommended guidelines (with modifications) for NTD mapping in the WHO AFRO Region [28].

The methods of data collection are described in Sanneh *et al* [26]. Demographic information and water contact behaviour were recorded for each participant (S1 Questionnaire). In the field, each urine sample upon collection from the participant, was observed for visible blood in urine (macrohaematuria). A urine dipstick (Hemastix) was then dip in the samples to investigate microhaematuria levels. After the haematuria testing, each urine sample was well shaken, and 10 ml of urine passed through the urine filtration kit and the filter transferred to a slide for the examination of terminally spined *S*. *heamatobium* eggs, using field microscopes. Back in the laboratory, two Kato-Katz slides were prepared from each stool sample and were microscopically examined for laterally spined *S*. *mansoni* eggs [26].

We extracted the infection, demographic and water contact data of all the 2,018 participants and included them in the analysis of the current study.

### Data analysis

SPSS statistical software for windows, version 24 (SPSS, Chicago, IL, USA) was used for all statistical analysis and computation. Descriptive statistics were used to show the distribution of demographic variables, and cross-tabulation was used to demonstrate infection prevalence, with 95% confidence intervals estimated using the Wilson Score Method. Chi-square test was used to compare infection rates among regions, gender, and age categories. Since the egg count data were not normally distributed, non-parametric tests were used to compare infection intensity means for gender (Mann-Whitney Test) and age categories (Kruskal-Wallis Test). Chi-square was further used to test for association between haematuria (both macrohaematuria and microhaematuria) and *S*. *haematobium* infection while logistic regression was employed to assess relationships between *S*. *haematobium* infection status and presence of water bodies around schools. Logistic regression was further used to assess relationships between *S*. *haematobium* infection status and water contact behaviours of participants. A generalised linear model with negative binomial log link function was used to assess association between presence of water bodies around schools and egg intensity. Spearman’s correlation was employed to assess correlations between prevalence of schistosomiasis and egg intensity in schools. All maps were created using QGIS version 3.0 (Girona Open Source Geospatial Foundation).

## Results

### Demography

**Fig 2:**
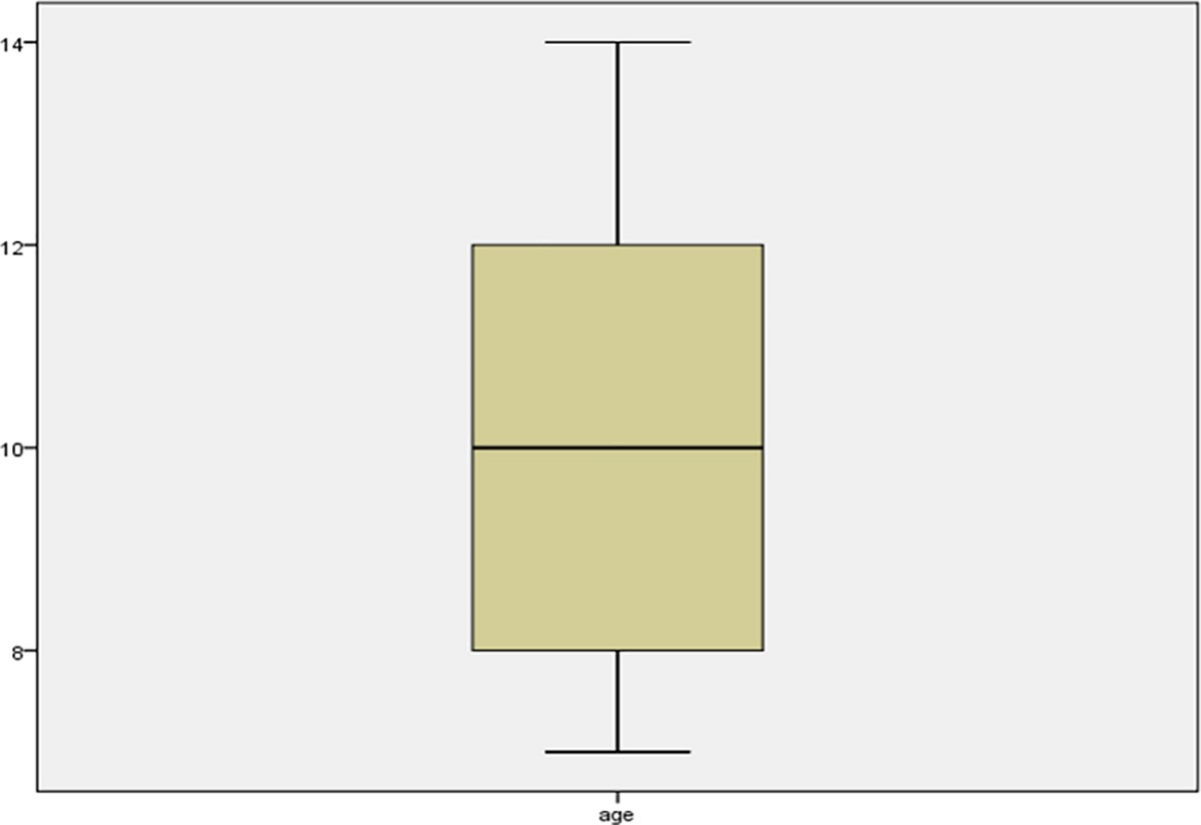
A box/whisker plot showing the age distribution of the study participants.

The study enrolled 2,018 primary school children, of which 1,010 were male, and 1,008 were female. Their ages ranged from 7 to 14 years. The median age (interquartile range) of the participants was 10 years (8 to 12 years). Fig 2 is a box/whisker plot showing the age distribution of the students enrolled in the study.

### Infection prevalence and intensity

The study revealed an overall prevalence of 10.2% (95% CI: 8.9–11.6%) for *S*. *haematobium* infection, with *Schistosoma mansoni* accounting for infection in just five students giving a prevalence of 0.3% (95% CI: 0.1–0.6%) among the sampled primary school children. A significant difference was observed in infection rate among regions for both *S*. *haematobium* (χ 2 = 279.958, df = 3, p < 0.001) and *S*. *mansoni* (χ 2 = 8.627, df = 3, p = 0.035). For *S*. *haematobium*, CRR was the most endemic of the 4 regions with a prevalence of 27.6% among the school children. URR, LRR and NBR had a prevalence of 12, 0.6, and 0%, respectively (Table 1). Four of the five students infected with *S*. *mansoni* were in CRR, and the fifth infected student was in URR. The rate of *S*. *haematobium* infection in boys (11.6%) was significantly higher than the rate of infection in girls (8.7%) across the four regions (χ 2 = 4.440, df = 1, p = 0.035). For *S*. *mansoni*, two males and three female students were infected with the intestinal worm. Prevalence of infection with *S*. *haematobium* was highest (10.9%) among the 10 to 12 years age category, followed by the 13 to 14 years age group (10.2%), then the 7 to 9 years category (9.5%) of the school children, see Table 1. However, these differences in infection rate among age groups were not statistically significant (χ 2 = 0.882, df = 2, p = 0.643). Four of the five individuals infected with *S*. *mansoni* were in the 10 to 12 years age group while the fifth person was in the youngest age group (7 to 9 years).

**Table 1:** Prevalence of urinary schistosomiasis (*S*. *haematobium* infection) among primary school children in 4 regions of The Gambia, 2015.

Variable	No. tested	No. positive	Prevalence, % (95% CI)
**Region**			
NBR	501	0	0.0 (0.0–0.8)
LRR	499	3	0.6 (0.2–1.8)
CRR	514	142	27.6 (23.9–31.7)
URR	502	60	12.0 (9.4–15.1)
**Gender**			
Male	1010	117	11.6 (9.8–13.7)
Female	1006	88	8.7 (7.2–10.7)
**Age category**			
7–9	953	91	9.5 (7.8–11.6)
10–12	750	82	10.9 (8.9–13.4)
13–14	313	32	10.2 (7.3–14.1)
**Total**	2,016*	205	10.2 (8.9–11.6)

*Two of the study participants have missing data for *S*. *haematobium* infection and were not included in this analysis, making the total number 2,016 participants instead of 2018.

**Fig 3:**
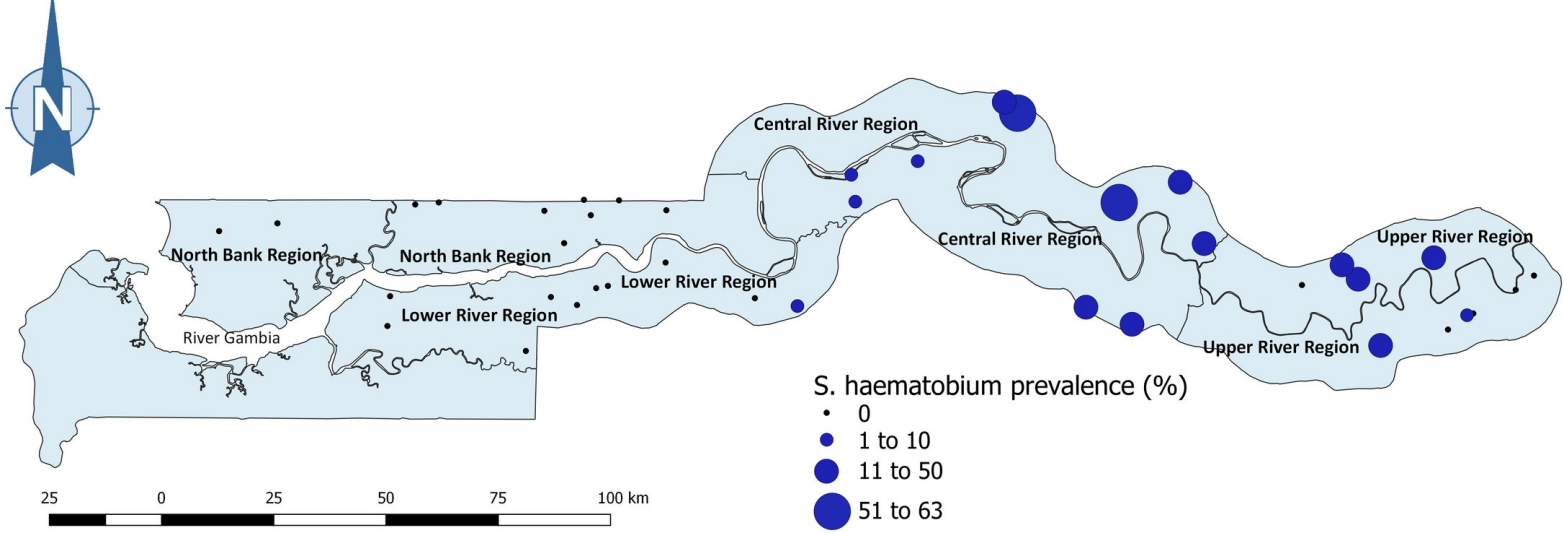
Prevalence of *S*. *haematobium* infection by school in 4 regions of The Gambia. **Ten schools were sampled in each region. Link to map base layer used in creating this figure:** https://data.humdata.org/dataset/gambia-administrative-boundaries.

In terms of school prevalence, at least one student was infected with *S*. *haematobium* in every school sampled in CRR (Fig 3). Schools in CRR had *S*. *haematobium* prevalence ranging from 5.7% to 63.0%, and the only two schools with an infection rate more than 50% (51.0% and 63.0%) were in CRR (Fig 3). Half of the schools sampled in URR had *S*. *haematobium* infection, with infection prevalence ranging from 1.9% to 38.0%. In LRR, *S*. *haematobium* infection was found in just one school with a prevalence of 6.0%, while no *S*. *haematobium* infection was found in any of the ten schools sampled in NBR (Fig 3). See S1 Table for *S*. *haematobium* prevalence of each school. Two of the four *S*. *mansoni*-infected students in CRR were in the same school, while the other two infected cases were in two different schools (one case each). The only school with *S*. *mansoni* infection in URR had just one student infected.

**Fig 4:**
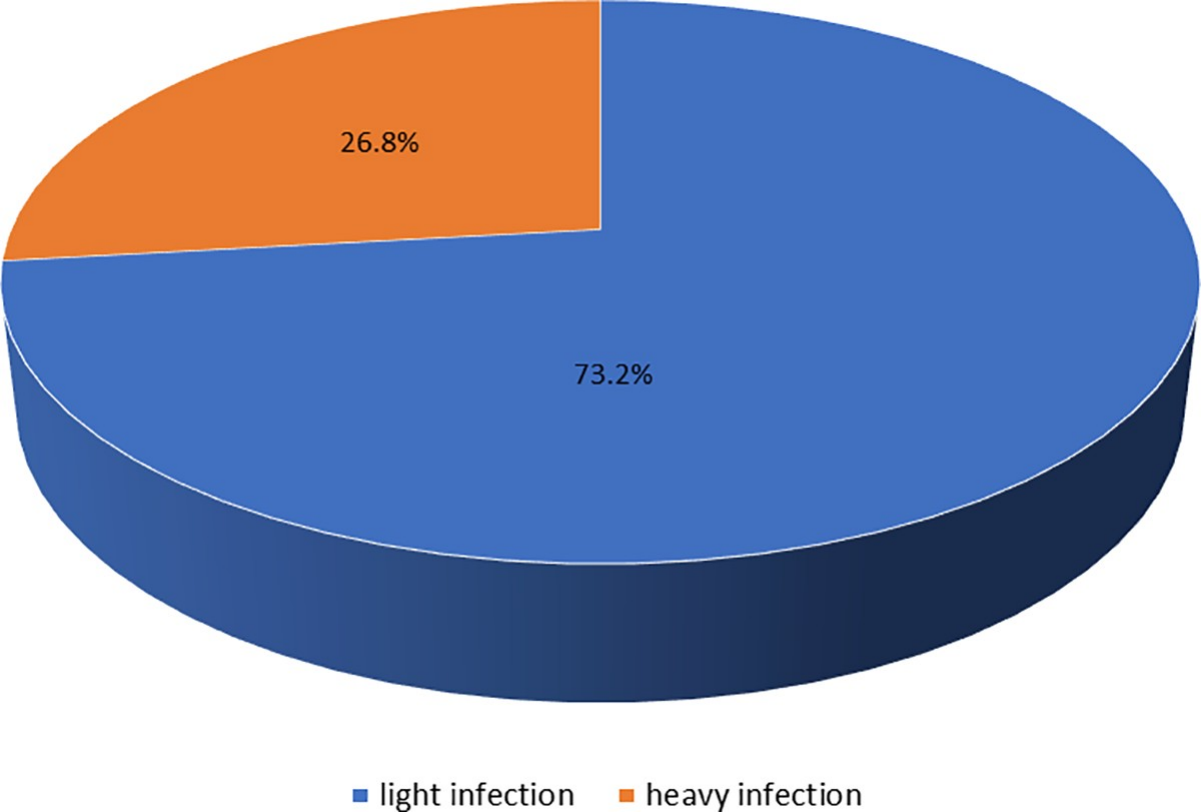
Infection intensity of *S*. *haematobium* (light against heavy infection) among children from 40 primary schools in 4 regions of The Gambia.

The mean (SD) egg count for *S*. *haematobium* was 6.15 (40.955). Of the 205 individuals infected with *S*. *haematobium*, 150 (73.2%) had ‘light infection’ (1–49 eggs/10 ml of urine), and 55 (26.8%) manifested ‘heavy infection’ (≥ 50 eggs/10 ml of urine), see Fig 4. All the participants infected with *S*. *mansoni*, presented ‘light infection’ intensity (< 400 eggs/g of faeces). Males had significantly higher infection intensity than females for *S*. *haematobium* (Mann-Whitney Test = 493170.0, p = 0.030). There was no significant difference in *S*. *haematobium* egg counts among age groups (Kruskal-Wallis Test, chi-square = 1.016, df = 2, p = 0.602). A Spearman’s correlation analysis found a positive correlation between prevalence of *S*. *haematobium* infection and egg intensity (r = 0.995, p < 0.001).

### Macro and microhaematuria

Overall, 10 (0.5%) individuals had visible blood in their urine or macrohaematuria, with 9 (90%) of them positive for *S*. *haematobium* eggs. Of the 2,006 participants negative for macrohaematuria, only 9.8% (196) had eggs in their urine (Table 2). Fisher’s Exact Test showed a significant association between macrohaematuria and the presence of *S*. *haematobium* eggs in urine (OR = 83.11, 95% Cl 10.48–659.46, p<0.001). For the dipstick method (microhaematuria), 363 (18.0%) individuals were positive overall. One hundred and fifty-three (74.6%) of the 205 individuals infected with *S*. *haematobium* were positive for microhaematuria, and 88.8% of the total 1,811 students that were not infected with urinary schistosomiasis showed no presence of microhaematuria in their urine (Table 2).

**Table 2:** Frequency of macro and microhaematuria among the studied school children, 2015.

	*S*. *haematobium* infection		
	Positive (%)	Negative (%)	Total (%)
**Macrohaematuria**			
Yes	9 (90.0%)	1 (10.0%)	10 (100%)
No	196 (9.8%)	1810 (90.2%)	2,006 (100%)
Total	205	1811	2016
**Microhaematuria**			
Yes	153 (42.1%)	210 (57.9%)	363 (100%)
No	52 (3.1)	1601 (96.9)	1,653 (100%)
Total	205	1811	2,016*

*Two of the study participants have missing data for *S*. *haematobium* infection status, which brings the total number to 2,016 instead of 2,018 participants.

### Risk factors

The univariate analysis showed that males were more likely to get infected with *S*. *haematobium* than females (OR = 1.37, 95% CI 1.02–1.83, p = 0.036), and no association was found between age category and urinary schistosomiasis infection (Table 3).

**Table 3:** Demographic variables as risk factors of urinary schistosomiasis infection among school children in 4 regions of The Gambia, 2015.

Variable	*S*. *haematobium* infection status			OR (95% CI)	p-value
	Total (%)	Positive (%)	Negative (%)		
**Gender**					
Male	1,010 (100)	117 (11.6)	893 (88.4)	1.37 (1.02–1.83)	0.036*
Female	1,006 (100)	88 (8.7)	918 (91.3)	1	
**Age category**					
7–9	953 (100)	91 (9.5)	862 (90.5)	1	
10–12	750 (100)	82 (10.9)	668(89.1)	1.16 (0.85–1.59)	0.348
13–14	313 (100)	32 (10.2)	281 (89.8)	1.08 (0.71–1.65)	0.727

*Statistical significance at p<0.05

OR = Odd Ratio

A generalised linear model with negative binomial log link function found a significant association between egg intensity and proximity of schools to water bodies (b = 0.429, 95% Cl = 0.318–0.541, p < 0.001). Egg intensity was higher in schools that had a fresh water body close by 15 minutes round-trip. Logistic regression found no significant association between *S*. *haematobium* infection status and presence of water bodies close by the schools (OR = 1.07, 95% Cl 0.76–1.50, p = 0.719. Water-contact behavioural risk factors such as washing clothes/dishes, fishing, fetching water, and crossing water bodies were not significantly associated with *S*. *haematobium* infection (see Table 4). Of the 205 individuals positive for *S*. *haematobium*, only 25 (12.2%) responded yes for bathing in freshwater bodies, 22 (10.7%) said yes for playing in freshwater bodies, and 15 (7.3%) said yes for swimming in freshwater bodies. Binary logistic regression analyses showed an unexpected association between urinary schistosomiasis infection and water-contact activities like bathing, playing, and swimming in water bodies. Instead of being risk for infection, bathing (OR = 0.59, 95% Cl 0.39–0.92, p = 0.019), playing (OR = 0.55, 95% Cl 0.35–0.88, p = 0.012) and swimming (OR = 0.52, 95% Cl 0.30–0.898, p = 0.019) were found to be less risk for *S*. *haematobium* infection, i.e. they were found to reduce the risk of infection with *S*. *haematobium* (Table 4). See S1 Data for the infection information and water contact responses of participants.

**Table 4:** Behavioural risk factors of *S*. *haematobium* infection in 4 regions of The Gambia, 2015.

Variable	*S*. *haematobium* infection status			OR (95% Cl)	p-value
	Total (%)	Positive (%)	Negative (%)		
**Water bodies**					
Yes	452 (100)	48 (10.6)	404 (89.4)	1.07 (0.76–1.50)	0.179
No	1564 (100)	157 (10.0)	1407 (90.0)	1	
**Bathing**					
Yes	368 (100)	25 (6.8)	343 (93.2)	0.59 (0.39–0.92)	0.019*
No	1,648 (100)	180 (10.9)	1468 (89.1)	1	
**Washing clothes/dishes**					
Yes	60 (100)	6 (10.0)	54 (90.0)	0.97 (0.42–2.31)	0.965
No	1,956 (100)	199 (10.2)	1,757 (89.8)	1	
**Fishing**					
Yes	69 (100)	5 (7.2)	64 (92.8)	0.68 (0.27–1.71)	0.417
No	1,947 (100)	200 (10.3)	1,747 (89.7)	1	
**Crossing water bodies**					
Yes	33 (100)	2 (6.1)	31 (93.9)	0.57 (0.13–2.38)	0.437
No	1,983 (100)	203 (10.2)	1,781 (89.8)	1	
**Fetching water**					
Yes	32 (100)	2 (6.3)	30 (93.8)	0.59 (0.14–2.47)	0.465
No	1,984 (100)	203 (10.2)	1,781 (89.8)	1	
**Playing**					
Yes	345 (100)	22 (6.4)	323 (93.6)	0.55 (0.35–0.88)	0.012*
No	1,671 (100)	183 (11.0)	1,488 (89.0)	1	
**Swimming**					
Yes	253 (100)	15 (5.9)	238 (94.1)	0.52 (0.30–0.898)	0.019*
No	1763 (100)	190 (10.8)	1573 (89.2)	1	

*Statistical significance at p<0.05

## Discussion

Human schistosomiasis was first reported in The Gambia in 1945 [29], but a more detailed account was given in 1954 by Duke and McCullough [20] on the distribution of the disease in Central River Region (CRR), then called McCarthy Island Division. Several studies on the disease followed over the next three decades until the mid-1980s [21,24,30,31]. After this period, efforts geared towards the control of schistosomiasis in the country (including prevalence studies and interventions) became limited. In 2014 when The Gambia took its first steps in setting up a schistosomiasis control programme, a knowledge gap on the distribution of the disease was found [25]. This study was conducted to provide information on the endemicity of the disease to help fill that knowledge gap.

The study assessed the prevalence and associated risk factors of schistosomiasis among primary school children in four of the five regions of the country. As reported by previous studies [20,21], urinary schistosomiasis was the predominant form of schistosomiasis seen in this study, with intestinal schistosomiasis observed in just 0.3% of the sampled school children investigated for infection. Of the numerous past studies on schistosomiasis in The Gambia, only one study reported the occurrence of intestinal schistosomiasis in the country [32]. Additionally, few or no cases of intestinal schistosomiasis have presented at hospitals and health facilities in recent times [25]. This led to the notion that *S*. *mansoni* does not exist in the country and that the few cases encountered in health facilities and studies might be imported cases. In this study, a follow-up on 2 of the five participants positive for *S*. *mansoni*, revealed they had a recent travel history. Prior to the study, the two students who are twins from the same household travelled to Guinea Conakry where *S*. *mansoni* is endemic [33,34].

The study found the highest prevalence of schistosomiasis among the school children in CRR and URR. This corroborates previous studies that found the eastern regions (represented by CRR and URR) to be the parts of the country endemic for urinary schistosomiasis [20,23,30,31]. In this study, CRR had a higher prevalence of *S*. *haematobium* than URR, with at least one student found infected in each of the ten schools sampled in the region. The River Gambia is fresh along both regions and has many tributaries (streams), locally called ‘bolongs,’ along these regions, which serve as sources of infection in the dry season [23]. Further away from the River Gambia, the landscape is a sandstone plateau with rocky, laterite iron pan [35]. When depressions formed in this laterite plateau, they hold rainwater and give rise to seasonal pools that become sources of infection in the rainy season [24,36,37]. Additionally, CRR has a huge irrigation project under the Ministry of Agriculture of The Gambia, with an irrigated area that stretches along many communities in the region engaged in rice farming. These factors might explain why the school children from these regions had the highest prevalence of urinary schistosomiasis. Conversely, the River Gambia is brackish in NBR and LRR, and there is no laterite plateau in these regions, which might be the reason for the zero and low infection rates among the school children in these regions.

Observations for both visible blood in urine (macrohaematuria) and non-visible blood in urine (microhaematuria) were found associated with presence of *S*. *haematobium* eggs in urine. Previous studies also found presence of blood in urine to correlate with presence of *S*. *haematobium* eggs, and it has been demonstrated as a good indicator of *S*. *haematobium* infection [13,17]. While not all haematuria are related to *S*. *haematobium* infection, presence of blood in urine provides an early defining indication of infection. The presence of blood in urine is caused by granulomatous inflammation resulting from the lodging of *S*. *haematobium* eggs in the bladder and urogenital system [2], and is increasingly becoming an important criteria for assessing *S*. *haematobium* infection in studies. Self reported blood in urine is even used to identify potential *S*. *haematobium* infection in people living in endemic areas [38]. Prevalence of *S*. *haematobium* was found to increase with increase in egg intensity. Egg intensity was also higher in schools that had a water body close by 15 minutes round-trip.

Previous studies have shown schistosomiasis prevalence to be higher in males than females [6,8,10,11,39], with males up to 16 times more likely of getting infected [6]. In this study, male students had a higher infection rate and had higher risk of getting urinary schistosomiasis than females. The context here is that ’male children’ are more likely to play in freshwater bodies and/or be responsible for carrying out important designated tasks such as watering livestock and other domesticated animals. Geleta *et al*. [11] found ‘having a parent as a farmer’ to be a predisposition for schistosomiasis while Amuta and Houmsou [8] found ‘being involved with irrigation’ as a risk factor for the disease. This study found no significant difference in *S*. *haematobium* infection prevalence among the different age groups and no association between age groups and *S*. *haematobium* infection. Many previous studies that involved school aged children made similar observations [9–11], while other studies reported the opposite [40,41].

Schistosomiasis is known to infect humans during water contact behaviours. Direct contact with open freshwater bodies through bathing, swimming, and wading is reported to be a risk factor of schistosomiasis [6,8,9,13]. In this study, however, for each water contact activity, less than 13% of the children infected with *S*. *haematobium* indicated that they were engaged in such water contact behaviour. From experience and our broad observations, this seemed an underrepresentation of their true water contact behaviours. Regression analysis of this data further indicated that children that engaged in some water contact activities (bathing, playing, and swimming) were less likely to get *S*. *haematobium* infection. A possible explanation is that the responses provided by the children are not a true representation of their water contact behaviour and activity, although the field teams ensured the children were comfortable throughout the interviews and were given gifts. While this finding did raise some questions–notably about response quality [42] around contact with water bodies, we are confident that it represented the response of the children interviewed. As most parents and teachers in these regions know about the disease, they will have advised their children against playing or bathing in these water bodies. For this reason, the children would naturally be uncomfortable disclosing contact with waterbodies for fear that they will be reprimanded by the teachers, parents or even us the researchers. The children may therefore have given inaccurate responses about their water contact behaviours.

Furthermore, if we explore the case of the children who are likely to be exposed to the water bodies by virtue of assumed responsibilities for carrying out activities such as watering livestock and other domesticated animals, an important potential risk factor is the proximity to the water source. Indeed, in a study conducted in Sudan by Hajissa and colleagues in 2018 [39], the authors identified the distance between children’s place of residence and the water source as an important determinant to the risk of *s*chistosomiasis infection in children. This factor was not assessed in the present study and is one of the limitations of the study. We however assessed the proximity of children’s schools to water bodies but found no significant association with schistosomiasis disease outcome. Children spend far more time away from school than in school. Additionally, a major source of schistosomiasis infection in The Gambia is seasonal pools which were non-existent during this study as the study was conducted in the dry season. These may explain the non association between proximity of schools to water bodies and *S*. *haematobium* infection status. Other potential important cofounders which could have shed more light on risk factors of infection if they were assessed in this study include parents’ occupation and snail density.

More innovative, collaborative Frameworks and approaches that ensure true response to questionnaires in studies involving children need to be developed and used, as children in early childhood tend to please the researcher or teacher [43]. Moreover, children are sometimes afraid to say something wrong or undesirable, especially in school-like settings [44]. We suggest that, to avoid anxiety in children, questions formulated for questionnaires and interviews should not resemble a school test, and children should be reassured that there is no wrong answer and that they will not be reprimanded for giving any particular answer. The questions should take into account cultural nuances which may serve as ‘unaccounted for’ confounders in researches involving children. Doing so will hopefully encourage honest feedback from children, who in some cases by virtue of associating researchers with ‘local authority’ are potentially affected by a ‘perceived power-dynamic hierarchy’.

Another point to make is that the The Taskforce for Global Health developed questionnaires we used to collect water contact data did not include activities like ‘being involved with irrigation farming’ and ‘watering livestock or domesticated animals in freshwater bodies.’ Yet, in rural Gambia, many children assist their parents in irrigation farming, as well as watering livestock and other domesticated animals. The lesson to draw from this is that protocols/frameworks should be tailored to country-specific or local needs as much as possible.

The findings of the study revealed information that was vital for the follow up interventions in the national schistosomiasis control programme of The Gambia. The prevalence data of the study revealed that nine districts (five in CRR, three in URR and one in LRR) in the study area were endemic for schistosomiasis and required chemotherapy. A follow up mass drug administration campaign was conducted in 2017 in these districts found eligible for chemotherapy, and 43, 013 people that included pre-school age children, school age children and adults were treated with 600mg single dose of praziquantel [45]. Although this study provided important information that guided mass chemotherapeutic interventions in 2017, new and continuous studies are still required to provide up to date prevalence estimates to guide subsequent rounds of MDA and other interventions of the control programme. These future studies should employ more advance diagnostic methods such as CCA and PCR, which are more sensitive than the traditional parasitological techniques used in this study. The studies should also focus on all aspects of the population, as the present study has a limitation that it sampled only primary school children. More studies on *S*. *mansoni* and its intermediate snail hosts are also required to describe intestinal schistosomiasis status in The Gambia.

## Conclusion

The study evaluated the prevalence and associated risk factors of schistosomiasis in primary school children in 4 regions of The Gambia (NBR, LRR, CRR, and URR) in 2015. Urine and stool samples were collected from 2,018 students and examined for *S*. *haematobium* and *S*. *mansoni* infections. 10.2% had *S*. *haematobium* infection, while 0.3% had *S*. *mansoni* infection. CRR was the most endemic region for *S*. *haematobium* infection, followed by URR, then LRR, and NBR had no infection. The prevalence of *S*. *haematobium* infection in boys was more than the prevalence in girls. No significant difference in infection rate was observed among the different age groups involved in the study. Boys had a higher risk of getting infected than girls, and presence of blood in urine was a good indicator of urinary schistosomiasis infection. Water contact activities such as bathing, playing and swimming were found to pose less risk for urinary schistosomiasis infection, suggesting untrue responses from children about their water contact behaviours. The findings of this study provide invaluable information on the endemicity of schistosomiasis in The Gambia that guided mass durg administration campaigns in the study area. More studies on *S*. *mansoni*, including investigation of its intermediate snail hosts, are required to establish the true status of intestinal schistosomiasis in the country.

## Supporting information

S1 Questionnaire Questionnaires used for data collection. (PDF)

S1 Table School prevalence and location coordinates. (DOCX)

S1 Data Infection status, egg count and water contact behaviour responses of participants. (XLSX)
