# Supplementary material for: Prevalence and risk factors of schistosomiasis among primary school children in four selected regions of The Gambia
Source: PLoS Negl Trop Dis. 2021 May 11;15(5):e0009380. doi: 10.1371/journal.pntd.0009380 (PMC8139473; doi:10.1371/journal.pntd.0009380)
Supplement: S1 Questionnaire — (PDF) [file pntd.0009380.s001.pdf]

Gambia AFRO Individual v3 (eng)

Variable Name	Question Text	Saved Value
meta	Hidden from user	
instanceID	Hidden from user	
Q1_Recorder	Enter Recorder ID	User entered integer
Q2_Diseases	Select disease(s) surveyed at this location	1 LF only<br>2 Oncho only<br>3 LF and Oncho<br>4 LF and Loa<br>5 Loa and Oncho<br>6 LF, Loa, and Oncho<br>7 Schisto and/or STH
Q3_Community_code1	Enter community/village code	User entered integer
Q4_Community_code2	Enter community/village code again	User entered integer
Q5_School_code1	Enter school code	User entered integer
Q6_School_code2	Enter school code again	User entered integer
Q7_Consent	Select enrollment type	1 Consent<br>2 Absent<br>9 Refused
Consent	Hidden from user	
Q7a_Name	Enter name	User entered text
Q8_Sex	Select sex	1 Male<br>2 Female
Q9_Age	Enter age	User entered integer
Q9a_Age_STH	Enter age	User entered integer
Q10a_Barcode	Barcode scan or manual entry?	1 Scan<br>2 Manual
Q10b_Scan	Scan barcode	User captured barcode

Q10c_Manual1	Enter barcode	User entered text
Q10d_Manual2	Enter barcode again	User entered text
Q11_How_long	How long have you lived in this community/village?	1 10+ years<br>2 5-9 years<br>3 1-4 years<br>4 Less than 1 year
LF	Hidden from user	
Q12_Bednet	Did you sleep under a bednet last night?	1 Yes<br>2 No
Q12a_ITN	Was the bednet treated with an insecticide when you obtained it?	1 Yes<br>2 No<br>7 Do not know
Prompt1	Ask individual the following questions about LF morbidity	User entered text
Q14_Lymphedema	Do you have lymphedema?	1 Yes<br>2 No
Q15_Lymphedema_upper	Do you have lymphedema in the upper limbs?	1 Yes<br>2 No
Q15a_Site	Observe patient. Select upper limb lymphedema site	1 Unilateral<br>2 Bilateral
Q16_Lymphedema_lower	Do you have lymphedema in the lower limbs?	1 Yes<br>2 No
Q16a_Site	Observe patient. Select lower limb lymphedema site	1 Unilateral<br>2 Bilateral
Q17_Lymphedema_breast	Do you have lymphedema in the breast?	1 Yes

		2 No
Q17a_Site	Select breast site	1 Unilateral<br>2 Bilateral
Q18_Lymphedema_genital	Do you have lymphedema in the vulva or penis?	1 Yes<br>2 No
Q19_Hydrocele	Do you have hydrocele?	1 Yes<br>2 No
Q20_Chyluria	Do you have chyluria?	1 Yes<br>2 No
Q20a_Chyluria_notes	Enter specific chyluria notes	User entered text
LFLoa	Hidden from user	
Prompt2	Ask individual the following questions about Loa loa	User entered text
Q21_Worms	Have you ever experienced or noticed worms move along the white of your eye?	1 Yes<br>2 No
Q22_Picture	Have you ever had the condition in this picture?	1 Yes<br>2 No
Q22a_Picture_freq	How many times have you experienced this over the past year?	User entered integer
Q22_Picture_last	How long ago did you last experience it (in months)?	User entered integer
Q23_Picture_days	How long did the worm last in your eye (in days)?	User entered integer
Q26_Nodules	Do you have nodules under the skin (not fixed and not painful)?	1 Yes<br>2 No
STH	Hidden from user	
Q28_School_urinate	When you are at school, where do you usually go to urinate?	1 In the school toilet<br>2 Around school compound

		3 Outside of school compound<br>4 In river/body of water<br>5 I wait/hold it<br>6 Other
Q28a_Urinate_other	If other, specify	User entered text
Q29_School_defecate	When you are at school, where do you usually go to defecate?	1 In the school toilet<br>2 Around school compound<br>3 Outside of school compound<br>4 In river/body of water<br>5 I wait/hold it<br>6 Other
Q29a_Defecate_other	If other, specify	User entered text
Water	Hidden from user	
Q30b_Bathing	Bathing	1 Yes<br>2 No
Q30c_Washing	Washing clothes/dishes	1 Yes<br>2 No
Q30d_Fishing	Fishing	1 Yes<br>2 No
Q30e_Crossing	Crossing water	1 Yes<br>2 No
Q30f_Fetching	Fetching water	1 Yes<br>2 No
Q30g_Playing	Playing	1 Yes

		2 No
Q30h_Swimming	Swimming	1 Yes<br>2 No
Q30j_No_activity	No activity	1 Yes<br>2 No
Q30a_None	No nearby water bodies	1 Yes<br>2 No
Q30k_Other	Other (specify)	User entered text
Q31_Notes	Additional notes	User entered text
Start_time	Hidden from user	Timestamp of form open
End_time	Hidden from user	Timestamp of form save

Gambia AFRO School WASH - Schisto STH v2 (eng)

Variable Name	Question Text	Saved Value
meta	Hidden from user	
instanceID	Hidden from user	
Q1_Recorder	Enter Recorder ID	User entered integer
Q2a_School1	Enter school code	User entered integer
Q2b_School2	Enter school code again	User entered integer
Prompt1	Ask teacher following questions	User entered text
Q3_Water_source	Is there a source of drinking water in the school?	1 Yes<br>2 No
Q4_Water_where	Where is the source of drinking water?	1 In the school<br>2 15 minutes or less round-trip from the school<br>3 More than 15 minutes round-trip from the school
Q5_Water_type	What type of water source?	1 Unprotected spring<br>2 Protected spring<br>3 Unprotected dug well<br>4 Protected dug well<br>5 Hand pump/tubewell/borehole<br>6 Surface water (river, dam, lake, stream, canal)<br>7 Public piped water/tap/standpipe<br>8 Rainwater collection<br>9 Plastic bag water<br>10 Bottle water<br>11 Other
Q5a_Water_other	If other, specify	User entered text
Q6_Water_bodies	Are there accessible water bodies close to the school?	1 Yes- 15 minutes of less round-trip<br>2 Yes - more than 15 minutes round-trip<br>3 No

Q7_Latrine	Is there a latrine in the school?	1 Yes<br>2 No
Q7a_Latrine_water	Is there water or tissue for use after defecating?	1 Always<br>2 Sometimes<br>3 Never
Latrine	Hidden from user	
Prompt2	Observe latrine	User entered text
Q8a_Latrine_type	What type of latrine is present?	1 Pit latrine without slab or open pit<br>2 Pit latrine with slab<br>3 Ventilated improved pit latrine (VIP)<br>4 Flush or pour flush toilet<br>5 Other
Q8b_Latrine_type_other	If other, specify	User entered text
Q8c_Latrine_condition	What is the condition of the latrine?	1 Poor (presence of flies, offensive odour and visible stool on floor, absence of roof/door)<br>2 Fair (presence of roof/door but dirty floor)<br>3 Moderate (clean, absence of roof/door)<br>4 Good (clean, odourless, no flies, presence of roof and door)<br>5 Excellent (Very clean, odourless, presence of door, roof, and availability of water)
Q8d_Latrine_water	Is there water or tissue for use after defecating?	1 Yes<br>2 No
Q8e_Latrine_hand	Is there provision for hand washing after latrine use?	1 Yes<br>2 No
Q8f_Latrine_hand_type	What type of hand washing facilities are available?	1 No water<br>2 Water only<br>3 Water and soap

		4 Water, soap, and non-disposable napkin<br>5 Water, soap, and disposable napkin<br>6 Other
Q8g_Latrine_hand_other	If other, specify	User entered text
Q9_Notes	Additional notes	User entered text
Start_time	Hidden from user	Timestamp of form open
End_time	Hidden from user	Timestamp of form save

Gambia AFRO Urine Results v2 (eng)

Variable Name	Question Text	Saved Value
meta	Hidden from user	
instanceID	Hidden from user	
Q1_Recorder	Enter Recorder ID	User entered integer
Q2a_Barcode	Barcode scan or manual?	1 Scan<br>2 Manual
Q2b_Scan	Enter barcode	User captured barcode
Q2c_Manual1	Enter barcode	User entered text
Q2d_Manual2	Enter barcode again	User entered text
Q3_Colour	Urine colour	1 Bloody<br>2 Amber/cloudy<br>3 Amber/clear<br>4 Pale yellow/clear<br>5 Pale yellow/cloudy
Q4_Test	Select test	1 Dipstick<br>2 CCA
Q4a_Dipstick_result	HEM urine dipstick result	1 Negative<br>2 Trace<br>3 +<br>4 ++<br>5 +++
Q4b_Dipstick_eggs	Number of Schistosoma haematobium eggs observed (filtration)	User entered integer
Q4c_CCA_result	CCA results	1 Positive<br>2 Negative<br>9 Not tested
Q4d_CCA_not	Why was test not done?	User entered text

Q5_Notes	Additional notes	User entered text
Start_time	Hidden from user	Timestamp of form open
End_time	Hidden from user	Timestamp of form save

Gambia AFRO Stool Results v2 (eng)

Variable Name	Question Text	Saved Value
meta	Hidden from user	
instanceID	Hidden from user	
Q1_Recorder	Enter Recorder ID	User entered integer
Q2a_Barcode	Barcode scan or manual?	1 Scan<br>2 Manual
Q2b_Scan	Scan Barcode	User captured barcode
Q2c_Manual1	Enter barcode	User entered text
Q2d_Manual2	Enter barcode again	User entered text
Q2e_Test	Select test	1 Kato Katz<br>2 PCR
KK	Hidden from user	
Q3_Ascaris	Ascaris lumbricoides detected?	1 Yes<br>2 No
Q3a_Ascaris_count	Enter number of Ascaris eggs observed	User entered integer
Q4_Trichuris	Trichuris trichuria detected?	1 Yes<br>2 No
Q4a_Trichuris_count	Enter number of Trichuris eggs observed	User entered integer
Q5_Hookworm	Hookworm detected?	1 Yes<br>2 No
Q5a_Hookworm_count	Enter number of Hookworm eggs observed	User entered integer
Q6_Schistosoma	Schistosoma mansoni detected?	1 Yes<br>2 No
Q6a_Schistosoma_count	Enter number of Schistosoma mansoni eggs observed	User entered integer
Q7_PCR	Was sample for PCR collected?	1 Yes

		2 No
Q7a_PCR_not	If not, why not?	User entered text
Q8_Notes	Additional notes	User entered text
Start_time	Hidden from user	Timestamp of form open
End_time	Hidden from user	Timestamp of form save
