# Supplementary material for: Prevalence and risk factors of schistosomiasis among primary school children in four selected regions of The Gambia
Source: PLoS Negl Trop Dis. 2021 May 11;15(5):e0009380. doi: 10.1371/journal.pntd.0009380 (PMC8139473; doi:10.1371/journal.pntd.0009380)
Supplement: S1 Table — (DOCX) [file pntd.0009380.s002.docx]

**S1 Table. School prevalence and location coordinates**

| **School_Name** | **Region** | ***S. haematobium* Prevalence (%)** | **Latitude** | **Longitude** |
| --- | --- | --- | --- | --- |
| Chagai wollof lower basic school | CRR | 63 | 13.58341 | -14.6659 |
| Jahanka Lower Basic School | CRR | 48 | 13.37859 | -14.7312 |
| Raneru Lower Basic School | CRR | 17.6 | 13.62318 | -14.5462 |
| Fory Lower Basic School | CRR | 11.8 | 13.50314 | -14.4993 |
| Njoren Basic Cycle School | CRR | 46 | 13.34511 | -14.6408 |
| Jamagan Lower Basic School | CRR | 5.9 | 13.58508 | -15.1838 |
| Kudang Lower Basic School | CRR | 7.8 | 13.66437 | -15.0614 |
| Jarreng Badala Lower Basic School | CRR | 5.7 | 13.63774 | -15.1918 |
| Pallol Lower Basic School | CRR | 51 | 13.7584 | -14.8652 |
| Jockul Ndowen Lower Basic School | CRR | 19.2 | 13.77997 | -14.8916 |
| Fatoto Lower Basic School | URR | 0 | 13.41254 | -13.8877 |
| Sotuma Lower Basic School | URR | 0 | 13.44038 | -13.8518 |
| Murreh Kunda Lower Basic School | URR | 24 | 13.47533 | -14.0485 |
| Suduwol Basic Cycle School | URR | 0 | 13.38153 | -13.9588 |
| Kiskis Lower Basic School | URR | 38 | 13.30364 | -14.1529 |
| Njayel Lower Basic School | URR | 0 | 13.33473 | -14.0206 |
| Koli Bantang Lower Basic School | URR | 34 | 13.43335 | -14.1971 |
| Touba Woppa Lower Basic School | URR | 22 | 13.46138 | -14.2286 |
| Sare Alpha Lower Basic School | URR | 1.9 | 13.38153 | -13.9588 |
| Kuwonku Lower Basic School | URR | 0 | 13.42195 | -14.3072 |
| Pakalinding Lower Basic School | LRR | 0 | 13.46594 | -15.5562 |
| Badumeh Kuta Lower Basic School | LRR | 0 | 13.39619 | -15.381 |
| Dongoroba Lower Basic School | LRR | 6 | 13.38078 | -15.2975 |
| Nema kuta Basic Cycle School | LRR | 0 | 13.41579 | -15.6924 |
| Kolior Lower Basic School | LRR | 0 | 13.42018 | -15.6694 |
| Sibito Lower Basic School | LRR | 0 | 13.38273 | -15.7301 |
| Bumari Lower Basic School | LRR | 0 | 13.39839 | -15.7814 |
| Kuyang Lower Basic School | LRR | 0 | 13.34174 | -16.1021 |
| Jouli Lower Basic School | LRR | 0 | 13.40005 | -16.0969 |
| Nioro Jattaba Lower Basic School | LRR | 0 | 13.29287 | -15.8305 |
| Challa Lower Basic School | NBR | 0 | 13.56832 | -15.5547 |
| Ngerr Lower Basic School | NBR | 0 | 13.58771 | -15.6475 |
| Yallal Tankonjala Lower Basic School | NBR | 0 | 13.55862 | -15.703 |
| Katchang Lower Basic School | NBR | 0 | 13.50389 | -15.7553 |
| Konteh kunda Lower Basic School | NBR | 0 | 13.56722 | -15.7942 |
| Kerr Ndongo Lower Basic School | NBR | 0 | 13.58864 | -15.7165 |
| Kerr Ardo Lower Basic School | NBR | 0 | 13.57961 | -16.0476 |
| Mbamori Kunda Lower Basic School | NBR | 0 | 13.5836 | -16.0015 |
| Ndungu Kebbeh Basic Cycle School | NBR | 0 | 13.5427 | -16.3181 |
| Ndofan Lower Basic School | NBR | 0 | 13.52746 | -16.4325 |
